# Glucoamylase of *Caulobacter crescentus* CB15: cloning and expression in *Escherichia coli* and functional identification

**DOI:** 10.1186/2191-0855-4-5

**Published:** 2014-01-27

**Authors:** Masayoshi Sakaguchi, Yudai Matsushima, Toshiyuki Nankumo, Junichi Seino, Satoshi Miyakawa, Shotaro Honda, Yasusato Sugahara, Fumitaka Oyama, Masao Kawakita

**Affiliations:** 1 Department of Applied Chemistry, Kogakuin University, 2,665-1 Nakano-cho, Hachioji, Tokyo 192-0015, Japan; 2 Translational Medical Research Center, Tokyo Metropolitan Institute of Medical Science, 2-1-6 Kami-kitazawa, Setagaya-ku, Tokyo 156-8506, Japan

**Keywords:** Cloning, Expression, Thermolabile glucoamylase, Characterization, Subsite affinity, Inhibition

## Abstract

The biochemical properties of the maltodextrin-hydrolyzing enzymes of cold-tolerant proteobacterium *Caulobacter crescentus* CB15 remain to be elucidated, although whose maltodextrin transport systems were well investigated. We cloned the putative glucoamylase of *C. crescentus* CB15 (CauloGA) gene. The CauloGA gene product that was expressed in *E. coli* was prone to forming inclusion bodies; however, most of the gene product was expressed in a soluble and active form when it was expressed as a fusion protein with *Staphylococcus* Protein A. The fusion protein was purified using an IgG Sepharose column and was identified as the active GA. The optimum temperature and pH for the activity of this GA toward maltotriose as a substrate were approximately 40°C and 5.0, respectively, and a differential scanning fluorimetry (DSF) analysis revealed that the melting temperature (*T*_m_) of CauloGA was 42.9°C. The kinetic analyses with maltotriose and other maltodextrins as the substrates indicated that CauloGA has higher *k*_cat_ and smaller *K*_m_ values at 30°C with both substrates compared with other GAs at lower substrate concentration. However, the enzyme activities toward the substrates decreased as the substrate concentrations increased at concentrations higher than approximately 10-fold the *K*_m_. The function-based identification of thermolabile *Caulobacter* GA contributes to the understanding of the maltodextrin-degradation system of *C. crescentus* as well as the bacterial GA’s function-structure relationship.

## Introduction

*Caulobacter crescentus* is a Gram-negative bacterium that can live in oligotrophic environments (Poindexter 1964). In *Caulobacter* sp. two groups of polysaccharide-hydrolyzing enzymes were reported to date: the cellulases including endoglucanase, exoglucanase and β-(1 → 4)-glucosidase (Song et al. 2013), and the β-xylosidases, xylosidase I and II (Graciano et al. 2012; Corrêa et al. 2012). In addition, the maltodextrin transport protein, MalA (*cc2287* gene product), was induced by either maltose or maltodextrins, and transported maltodextrins ranging from maltose to maltopentaose (Neugebauer et al. 2005). Investigations of the metabolism of maltodextrins including starch and of the enzymes involved in the maltodextrin metabolism are definitely required, and outcome from such investigations would help in understanding the carbohydrate metabolism of *Caulobacter*.

In the malA gene cluster, there are six genes, *cc2282*, *cc2283*, *cc2284*, *cc2285*, *cc2286* and *cc2287* that are also described as MalS, MalY, MalI, an α-amylase family protein, another α-amylase family protein and MalA, resepectively (Neugebauer et al. 2005; Lohmiller et al. 2008). Nierman et al. (2001) who reported the complete genomic sequence of *C. crescentus* CB15, also known as *Caulobacter vibrioides*, tentatively assigned the *cc2282* gene to a putative glucoamylase (GA) gene based on similarity with other GA genes without functional evidence.

Glucoamylase (GA) is classified to the glycoside hydrolase (GH) family 15 in the CAZy database (carbohydrate active enzymes database, http://www.cazy.org/). GA produces glucose from starch and is important in industry. This enzyme releases β-D-glucose from the non-reducing end of starch, glycogen and related oligo- and polysaccharides. GA shows a strong preference for the α-(1 → 4)-linkages over the α-(1 → 6)-linkages. Eukaryotic GAs, particularly *Aspergillus* GA, have been extensively investigated (Coutinho and Reilly 1997; Kumar and Satyanarayana 2009). Bacterial GAs have also been studied, though less extensively than the fungal enzymes. Despite the similarity in the catalytic function and the overall structure of the catalytic domains, the similarity of the amino acid sequences is low between eukaryotic GAs and bacterial GAs. The catalytic properties of GA from thermophilic Clostridia were studied in considerable detail (Ohnishi et al. 1992; Ohnishi et al. 1994; Ducki et al. 1998; Ganghofner et al. 1998; Aleshin et al. 2003), and the three-dimensional structure of *Thermoanaerobacterium thermosaccharolyticum* GA (TtGA) was determined (Aleshin et al. 2003). Properties of GAs from thermophilic archaea were also reported (Uotsu-Tomita et al. 2001; Serour and Antranikian 2002; Dock et al. 2008, Kim et al. 2004).

The deduced amino acid sequence of the *cc2282* gene showed 47% similarity to that of both TtGA and *Clostridium* sp. G0005 GA (CGA, ClostGA), and the gene is registered in the CAZy database as CcGA (CauloGA) which belongs to the GH 15 family. However, the assignment remains tentative, since the gene product has not been functionally characterized, and since the deduced amino acid sequence showed 46% similarity to that of an N-terminal and a catalytic domains of *Arthrobacter globiformis* I42 glucodextranase (AgGDase), a GH 15 family enzyme (Mizuno et al. 2004). It is therefore important to examine whether the gene product in fact represents GA. The conclusive identification of *Caulobacter* GA may contribute to the understanding of the maltodextrin-degradation system of *C. crescentus* as well as the bacterial GA’s function-structure relationship by adding a thermolabile member to those well characterized thermophilic group members. In this report, we cloned the putative GA gene (*cc2282*) from *C. crescentus* CB15, expressed it in *E. coli* and identified the gene product as a GA from *Caulobacter* (CauloGA) based on its functional properties.

## Materials and methods

### Bacterial strains and growth media

*Caulobacter crescentus* CB15 (ATCC No. 19089) was obtained from the American Type Culture Collection (ATCC). The *E. coli* strains used as the expression hosts and the gene engineering hosts were BL21 (DE3), DH5α, HB101, JM109, MV1184, Origami (DE3) and Tuner (DE3).

LB medium (1% Bacto Tryptone, 0.5% Bacto Yeast extract, 1% NaCl, pH 7.0) was used for culturing *E. coli* in the genetic engineering and in the preculture for protein expression. The solid medium contained 1.5% agar. Ampicillin (50 μg/ml) was added to the medium as needed.

### Gene cloning and chemical reagents

Genetic engineering experiments were performed according to the procedure described by Sambrook and Russell (2001). The enzymes used for genetic engineering were purchased from TAKARA BIO INC. (Shiga, Japan) and used according to the manufacturer’s instructions. Bacto Tryptone, Bacto Yeast extract and peptone were purchased from Becton, Dickinson and Company (Franklin Lakes, NJ, USA). Other reagents and oligosaccharides used as substrates were of the highest quality available from Wako Pure Chemicals (Osaka, Japan) and Sigma-Aldrich (St. Louis, MO, USA), unless otherwise specified.

### Cloning of the GA gene from *C. crescentus* CB15 and construction of the expression plasmids

*C. crescentus* CB15 was grown in Caulobacter medium (peptone, 2.0 g; yeast extract, 1.0 g; MgSO_4_•7H_2_O, 0.2 g in 1 liter) at 30°C for 2–3 days, and the genomic DNA was extracted using ISOPLANT II (Nippon Gene, Tokyo, Japan).

PCR was performed as follows: 1 cycle at 94°C for 2 min followed by 30 cycles of the sequence at 94°C for 15 s, 63°C for 30 s and 68°C for 2.5 min were performed, using 25 ng of the genomic DNA as a template, with the forward primer 5′-CGC*GGATCC*GCGATGCGCACGTTGAAAAC-3′, and the reverse primer 5′-G*GAATTC*CTAGCGCGCGTACCGCGC-3′ (the BamHI and EcoRI restriction sites are underlined), and the PCR product was cloned into the BamHI and EcoRI sites of pUC119 vector to give pUC119-CauloGA. Next, PCR was performed using the pUC119-CauloGA as a template, with the forward primer 2, 5′-GC*GAATTC*GGCGCCTACGACCTGGGCCTATTCG-3′, and the reverse primer 2, 5′-CG*GGATCC*CTAGCGCGCGTACCGCGCCTTTACGGG-3′ (the EcoRI and BamHI sites, respectively, are underlined), to remove a putative signal peptide sequence that was predicted by SignalP (http://www.cbs.dtu.dk/services/SignalP/). The amplified PCR product was cloned into the EcoRI and BamHI site of the pEZZ18 vector (GE Healthcare, Tokyo, Japan) to yield the expression vector, pEZZ18-CauloGA. Expression vectors, pCold I-CauloGA and pCold TF-CauloGA, were constructed similarly using the pCold vectors (TAKARA) and pUC119-CauloGA plasmid. The nucleotide sequences were confirmed with the Applied Biosystems 3130 Genetic Analyzer (Applied Biosystems, Foster City, CA, USA).

The nucleotide sequence of CauloGA is available in the DDBJ/EMBL/GenBank database under the accession number AB813000.

### Expression, purification and activity measurement of CauloGA

The *E. coli* that was transformed with the pEZZ18-CauloGA expression plasmid was precultured overnight in LB medium at 30°C, and the preculture was inoculated in fresh 2 × YT-medium (1.6% Bacto Tryptone, 1% Bacto Yeast extract, 0.5% NaCl, pH 7.0) at 30°C for 24 h, harvested by centrifugation, suspended in 20 mM Tris-HCl (pH 7.0) containing 0.5 M NaCl and sonicated on ice using a Ultrasonic disruptor UD-201 (TOMY SEIKO, Tokyo, Japan). The supernatant obtained from the crude extract by centrifugation for 20 min at 20,000 × *g* was adsorbed onto an IgG Sepharose column (GE Healthcare, Tokyo, Japan) that was equilibrated with TS buffer [50 mM Tris-HCl (pH 7.6) containing 150 mM NaCl]. The bound fusion proteins were eluted with TS buffer containing 40% (v/v) 1,4-dioxan (Kumari and Gupta 2012). The purity of CauloGA was confirmed by 9% SDS-PAGE (Laemmli 1970). The fraction containing the active enzyme was dialyzed against 20 mM Tris-HCl, 0.5 M NaCl (pH 7.0), and the purified protein was stored at 4°C.

The enzyme activity was measured at 40°C in 50 mM acetate buffer (pH 5.0) with 0.9 mM maltotriose as a substrate unless otherwise specified. The enzyme solution (50 μl) was added to 450 μl of the substrate solution, and the reaction mixture was incubated for an appropriate period. To halt the reaction, 50 μl aliquots of the mixture were removed from the reaction mixture and mixed with 450 μl of the stop solution, 1 M Tris-HCl (pH 7.0), at appropriate intervals, and the initial rate was estimated from the initial slope of the reaction time course. The amount of glucose liberated from the substrate was determined with the F-kit D-glucose (Roche Diagnostics Gmbh, Mannheim, Germany) using the glucose standard curve as a reference. One unit of GA was defined as the amount of the enzyme that liberates 1 μmol of glucose from the substrate in 1 minute. The protein concentration was measured using the micro-assay method (Bio-Rad Laboratories, Hercules, CA, USA), which is based on the Bradford method (Bradford 1976), using bovine serum albumin as a standard.

### Polyclonal antibody against the CauloGA gene product expressed in *E. coli* as an inclusion body

The (His)_6_-tagged CauloGA was expressed in *E. coli* as an inclusion body using the pCold I vector system (TAKARA). The inclusion bodies were dissolved in 20 mM Tris-HCl containing 6 M guanidine hydrochloride, and the denatured CauloGA protein was purified on HisTrap™HP (GE Healthcare) and the purified denatured CauloGA was used as the antigen to raise the rabbit anti- CauloGA antibody that was prepared by TAKARA.

### Western blotting analysis of CauloGA

The CauloGA expressed in *E. coli* using the pEZZ18-CauloGA expression plasmid was detected according to the procedure described in a previous report (Takeshima-Futagami et al. 2012). The CauloGA protein was detected using anti-CauloGA antibody and the HRP-conjugated goat anti-rabbit IgG (H + L) antibody (Invitrogen, Carlsbad, CA, USA) as the primary and the secondary antibodies, respectively. The bound antibodies were detected using the SuperSignal West Dura Trial Kit (Thermo Scientific, Rockford, IL, USA).

### Determination of the anomer type of the products of the CauloGA reaction

Maltotriose as a substrate was dissolved in D_2_O containing 50 mM 2-morpholinoethanesulfonic acid (MES)-NaOH (pH 6.0). The enzyme was mixed with 4.5 mM maltotriose and incubated at 30°C. At the appropriate reaction time, the ^1^H-NMR spectra were measured using a JNM-ECX-400 (JEOL, Tokyo, Japan) spectrometer. The chemical shifts were referenced to 2,2-dimethyl-2-silapentane-5-sulfonate (DSS) (Cambridge Isotope Laboratories, Inc., Andover, MA, USA), as the internal standard.

### Measurement of temperature- and pH-dependence of the activity, heat stability and pH stability of CauloGA

The temperature-dependence of the CauloGA activity was examined by measuring the initial rate as described above, at 15, 20, 25, 30, 35, 40 and 45°C. To determine the heat stability of CauloGA, the enzyme solution was incubated at various temperatures (4-60°C) for 60 min and cooled quickly, and the remaining activity was measured. The pH-dependence of the CauloGA activity was determined by measuring the initial rate at different pH values, ranging from pH 3.5 to 11, using 0.9 mM maltotriose as a substrate in various buffers as follows: 50 mM acetate buffer (pH 3.5-6.0), 50 mM MES-NaOH buffer (pH 5.0-7.0), 50 mM Tris-HCl buffer (pH 7.0-9.0) or 50 mM carbonate-NaOH buffer (pH 10-11). To study the pH-stability of CauloGA, the enzyme solution was placed in various buffers (pH 3.5-11) at 4°C for 60 min, and the remaining activity was measured.

### Differential scanning fluorimetry (DSF) analysis

The experiment was performed using a Real Time PCR System (Mx3005p, Agilent Technologies, Santa Clara, CA, USA) and MxPro Software (Agilent Technologies). Tubes containing a mixture of 39 μl of a protein solution and 1 μl of Sypro Orange (Invitrogen) diluted to 100-fold with dimethyl sulfoxide were set in the PCR instrument and were subjected to the temperature scan at 1°C min^-1^ from 25°C to 95°C. The filter configurations were customized to accommodate the optimal excitation and emission wavelengths for Sypro Orange (Ex: 492/Em: 610 nm). The inflection points of the transition curve, indicating the melting temperatures (*T*_m_) of the protein, were estimated from the sigmoidal curves of the fluorescence intensity using the Boltzmann equation and a graphics software package (DeltaGraph ver. 6, Nihon Poladigital K.K., Tokyo, Japan), according to the method of Niesen et al. (2007).

## Results

### Cloning of the CauloGA gene from *Caulobacter crescentus* CB15 and its expression in the soluble form in *E. coli*

To obtain the CauloGA gene (the *cc2282* genomic region) from *C. crescentus* CB15, the forward and reverse primers were designed based on the report by Nierman et al. (2001). The nucleotide sequence of the cloned gene revealed two T for C substitutions at the positions 945 and 1,656 compared with the sequence reported by Nierman et al. (2001), but the deduced amino acid sequence was not affected by these base substitutions (Additional file 1: Figure S1).

Despite attempts to obtain an active CauloGA protein using pET vectors (*e.g.* pET21, pET28 or pET32; Novagen, Madison, WI, USA) and chaperon-coexpression vectors (TAKARA) together with various *E. coli* hosts, CauloGA was not obtained in a soluble form. When we tested the pCold TF vector system (TAKARA), the CauloGA protein was expressed in a soluble form but was completely inactive in hydrolyzing maltotriose. Although most of the expressed CauloGA protein formed inclusion bodies using the pCold I vector, a small amount of CauloGA protein was recovered in the soluble fraction in DH5α and JM109, and the protein was proved to be catalytically active. This provides the rationale for using the insoluble CauloGA protein in inclusion bodies as an antigen to raise an anti-CauloGA antibody.

**Figure 1:**
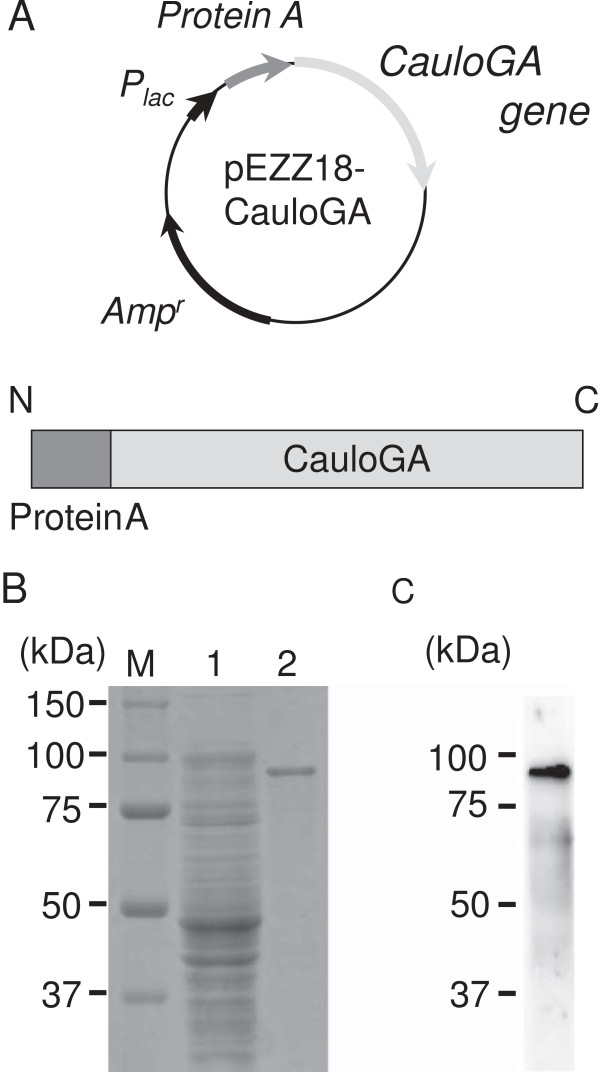
**The plasmid map and a schematic representation (A), the SDS-PAGE (B) and the western blotting analysis (C) of the Protein A-CauloGA fusion protein. (A)** The plasmid map of pEZZ18-CauloGA (upper) and a schematic representation of the Protein A-CauloGA fusion protein (lower). The construction of the plasmid is detailed in Materials and Methods. *P*lac, *Protein A* and *Amp*r denote the lac promoter, Protein A and the ampicillin-resistant genes of the pEZZ18 vector, respectively. The gray arrow represents the CauloGA gene. The dark and light shaded boxes represent Protein A (synthetic ZZ domain) and CauloGA, respectively. N and C indicate the N- and C- termini of the protein. **(B)** The SDS-PAGE analysis of the crude cell extract and the IgG Sepharose-purified CauloGA. Lane M, molecular weight marker (Precision Plus Protein™ Standards, Bio-Rad laboratories); lane 1, crude extract, supernatant from cell homogenates; and lane 2*,* purified CauloGA eluted from IgG Sepharose. The numbers in the margin represent the molecular masses (kDa) of the proteins in the molecular weight marker. **(C)** The Western blotting analysis of the purified CauloGA. The numbers represent the molecular masses (kDa) of the proteins in the molecular weight marker.

Finally, the CauloGA gene was recloned into the pEZZ18 vector, and active CauloGA was obtained in the soluble form in *E. coli* HB101 cells without IPTG induction at 30°C. In this system, CauloGA was available as a fusion enzyme in which *Staphylococcus* Protein A was fused at the N-terminal end of the CauloGA (Figure 1A). The fusion protein, ProteinA-CauloGA, was soluble, and the maltotriose-hydrolyzing activity was detected entirely in the cell homogenates. The expressed ProteinA-CauloGA fusion protein was purified by IgG Sepharose to give a single band upon SDS-PAGE/Western blotting analysis (Figures 1B and 1C).

### Analysis of the CauloGA reaction products

**Figure 2:**
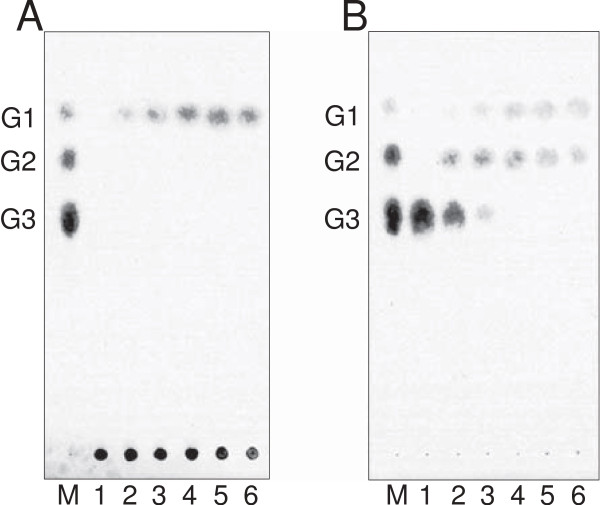
**Analysis of the CauloGA reaction products with starch (0.45%) (A) and maltotriose (4.5 mM) (B) as the substrates.** The reaction was performed at 30°C in 50 mM acetate buffer (pH 4.5), halted by boiling, and the products were analyzed by thin-layer chromatography (TLC) on a Silica gel 60 F_254_ (Merck, Whitehouse Station, NJ, USA) with 1-butanol/ethanol/water (5:5:2, v/v/v) as the solvent, and the carbohydrate spots were detected as chars by spraying the H_2_SO_4_/methanol (5:95, v/v) solution. G1, G2 and G3 glucose, maltose and maltotriose, respectively. Lane M, glucose standard; lane 1, no enzyme added; lane 2 to 6, the products obtained after hydrolysis for 5 min, 10 min, 15 min, 30 min and 60 min, respectively.

Figure 2 shows the thin-layer chromatogram of the products that were released by the CauloGA using soluble starch (Figure 2A) and maltotriose (Figure 2B) as the substrates. Glucose was the sole product of the starch hydrolysis by the CauloGA, and the amount of glucose increased as a function of the reaction time. With maltotriose as the substrate, glucose and maltose were initially produced, and then, maltose was further hydrolyzed to yield glucose. These results suggested that CauloGA behaved as an exo-glucosyl hydrolase.

The anomer type of the products released from maltotriose by the CauloGA reaction was determined by ^1^H-NMR analysis. The intensities of both the β-H (4.6 ppm) and α-H (5.2 ppm) peaks increased with the reaction time, but the increase of the β-H (4.6 ppm) peak was more pronounced (Gloster et al. 2008). This is unlike the pattern obtained when α-glucosidase (Sigma-Aldrich) was used as a control, and we concluded that the CauloGA produced glucose from maltotriose in the form of the β-anomer (Additional file 2: Figure S2A and S2B).

### Effects of the temperature and pH on the CauloGA activity

**Figure 3:**
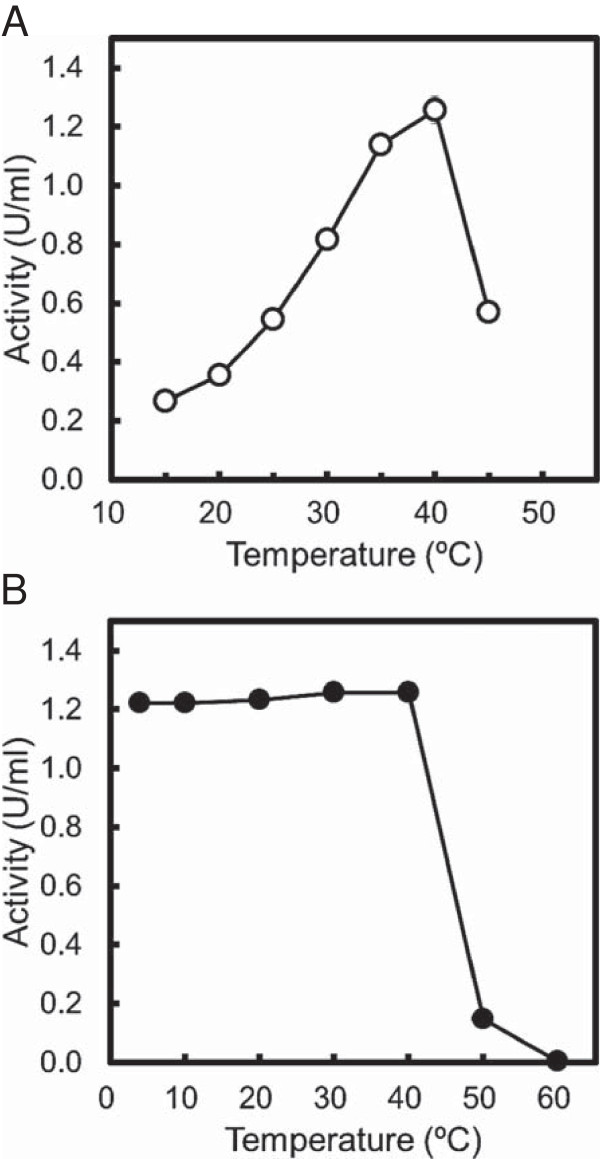
**Effects of temperature on the CauloGA activity (A, open circle) and stability (B, closed circle). (A)** The activity was measured at various temperatures (15-40°C). **(B)** CauloGA was incubated at various temperatures (4-60°C) for 60 min, and the remaining activity was measured. The values are represented as enzyme activity. Experiments were carried out in triplicate.

The dependence of CauloGA activity on the temperature was examined using 0.9 mM maltotriose in 50 mM acetate buffer (pH 5.0) at temperatures ranging from 10 to 45°C. Figure 3A shows that the activity of the CauloGA was maximal at approximately 40°C. The activity of the CauloGA that remained after heat treatment for 60 min in a range of 4 to 60°C was determined at 40°C with maltotriose as a substrate; the results are shown in Figure 3B. The activity of the CauloGA remained stable for 60 min at temperatures ranging from 4 to 40°C, but was almost completely lost at 50°C for 60 min. This result was consistent with the results of the DSF analysis of the CauloGA, which gave a *T*_m_ value of 42.9°C (Additional file 3: Figure S3).

**Figure 4:**
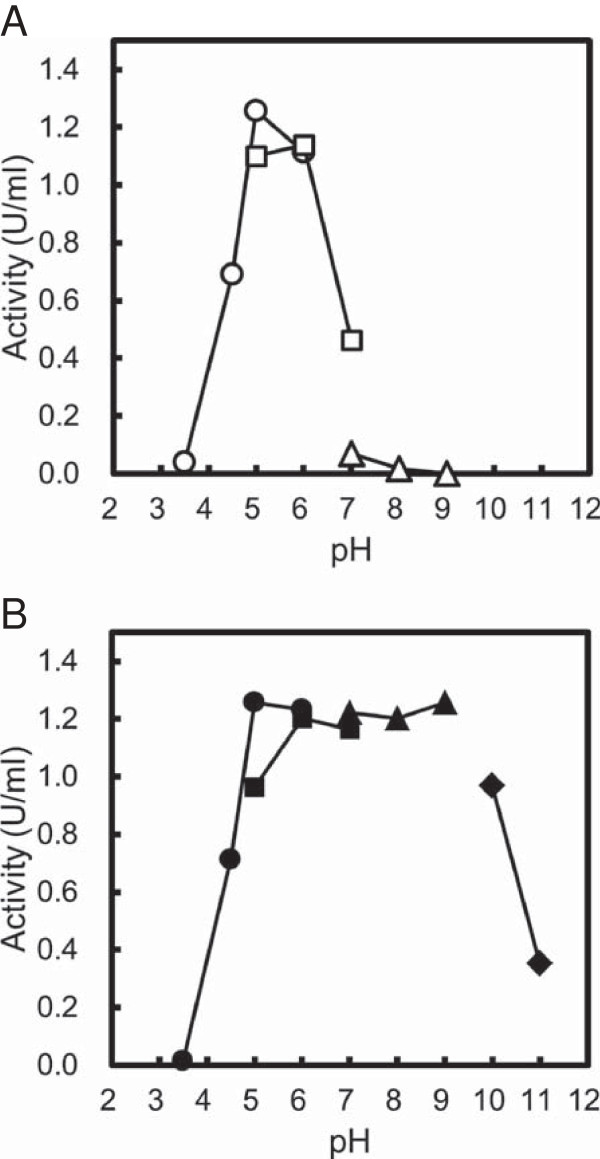
**Effects of pH on the CauloGA activity (A) and stability (B). (A)** The activity was measured at 40°C in various buffers at different pH values as follows: 50 mM acetate buffer (pH 3.5-6.0, open circle), 50 mM MES-NaOH buffer (pH 5.0-7.0, open square), or 50 mM Tris-HCl buffer (pH 7.0-9.0, open triangle). **(B)** CauloGA was incubated at 4°C for 60 min at various pH values as follows: 50 mM acetate buffer (pH 3.5-6.0, closed circle), 50 mM MES-NaOH buffer (pH 5.0-7.0, closed square) or 50 mM Tris-HCl buffer (pH 7.0-9.0, closed triangle), or 50 mM carbonate-NaOH buffer (pH 10-11, closed rhombus). The remaining activity was measured at 40°C and pH 5.0. The values are represented as enzyme activity. Experiments were carried out in triplicate.

Figure 4A illustrates the pH-dependence of the CauloGA activity. CauloGA was optimally active at pH 5 to 6 and remained active over a broad pH range, between pH 4.5 and pH 7.0, at which the enzyme demonstrated more than half of its maximal activity. Figure 4B illustrates the pH stability curve of CauloGA and shows that the enzyme retained its full activity after treatment for 60 min at pH 5.0-9.0.

### Substrate specificity and steady-state kinetics of CauloGA

**Figure 5:**
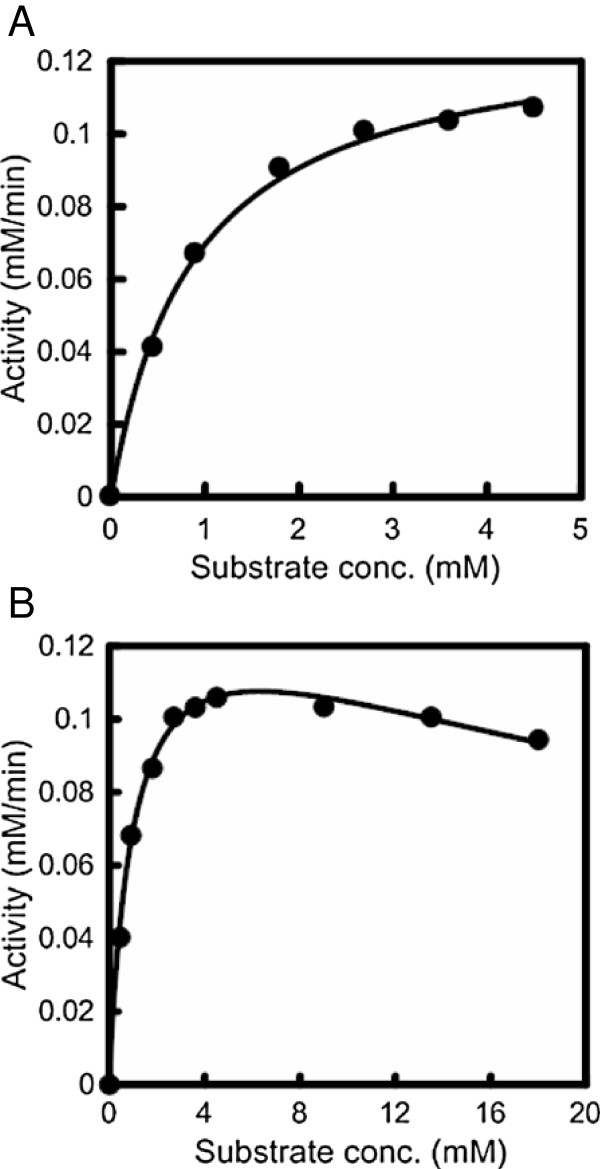
**Dependence of CauloGA activity on the maltose concentration.** The initial rate was measured at 30°C in 50 mM acetate buffer (pH 5.0) at various maltose concentrations: **(A)** 0–4.5 mM; **(B)** 0–18 mM. The kinetic parameters are listed in Table 2. Experiments were carried out in triplicate and the average initial rates are shown.

**Figure 6:**
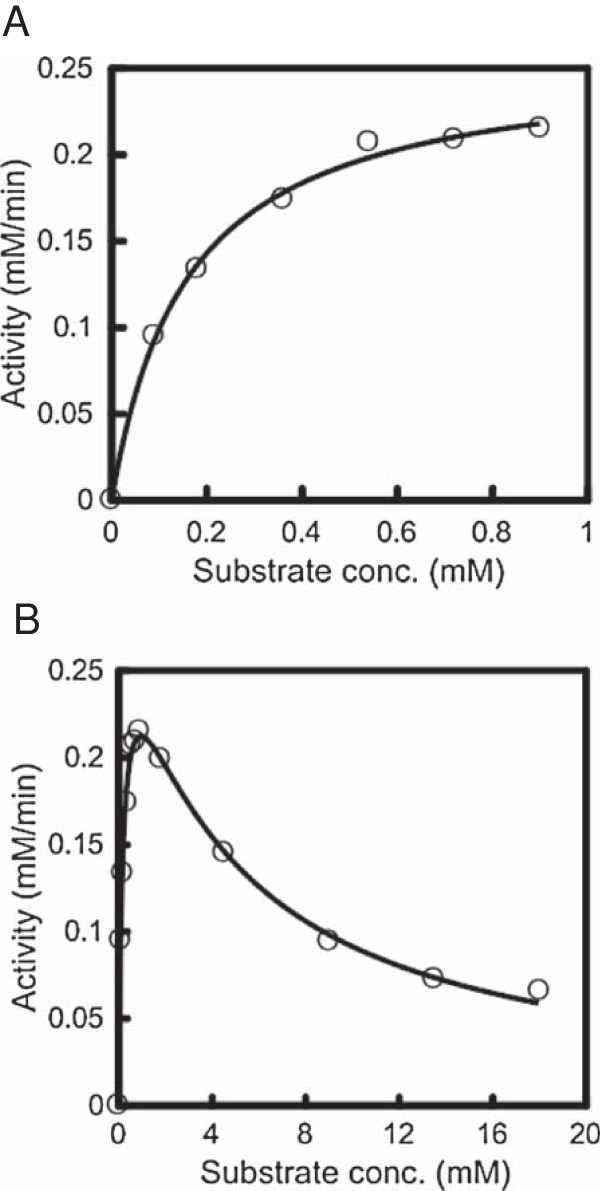
**Dependence of CauloGA activity on the maltotriose concentration.** The initial rate was measured at 30°C in 50 mM acetate buffer (pH 5.0) at various maltotriose concentrations: **(A)** 0–0.9 mM; **(B)** 0–18 mM. The kinetic parameters are listed in Table 2. Experiments were carried out in triplicate and the average initial rates are shown.

The specificity of the CauloGA activity was investigated at 40°C in 50 mM acetate buffer (pH 5.0) using various polysaccharides at 0.45% (w/v) substrate concentration as follows: starch, pullulan and dextran, and oligosaccharides; maltodextrins, isomaltotriose and panose. CauloGA actively hydrolyzed the substrates with α-(1 → 4)-glucosidic linkages at the non-reducing end, such as starch, maltose and maltotriose. Conversely, CauloGA showed little (if any) hydrolyzing activity on the substrates with α-(1 → 6)-glucosidic linkages, namely, pullulan, dextran and isomaltotriose (Table 1). CauloGA appeared characteristically active on α-(1 → 4)-glucosidic linkaged substrates like other bacterial and fungal GAs that have been examined for substrate specificity. However, the activities of CauloGA for maltodextrins were lower at 0.45% than 0.09% substrate concentration except for starch and maltose (Table 1). To understand the reason for this unusual behavior, we determined the steady-state kinetic parameters of CauloGA for various substrates using a DeltaGraph software by nonlinear regression. Figure 5A and Figure 6A show the initial rate of the CauloGA reaction at 30°C and pH 5.0 using maltose (0–4.5 mM) and maltotriose (0–0.9 mM) as substrates, and Table 2 summarizes the steady-state kinetic parameters for various substrates. In these lower substrate concentration ranges, the CauloGA reaction obeyed the Michaelis-Menten kinetic model. The estimated values of *k*_cat_ and *K*_m_ were 38.2 s^-1^ and 0.87 mM, and 150 s^-1^ and 0.16 mM, for maltose and maltotriose, respectively, assuming the molecular mass of 96 kDa that was deduced for the Protein A-CauloGA fusion. For other maltodextrins as substrates, the *K*_m_ values decreased to 0.03-0.05 mM. The CauloGA activity was significantly inhibited when the substrate concentration exceeded approximately 10-fold the *K*_m_ value. For instance, inhibition was pronounced at a concentration above 2 mM maltotriose (Figure 6B). The apparent substrate inhibition constant, *K*_i,_ for maltotriose was approximately 4.2 mM (Table 2). For other maltdextrins, the substrate inhibition was more pronounced and the apparent substrate inhibition constants were approximately 1 mM. Higher concentrations of maltose also slightly inhibited the CauloGA reaction, and the estimated *K*_i_ value was approximately 38 mM (Figure 5B, Table 2).

**Table 1:** Comparison of CauloGA activity for starch and maltodextrins

	**Substrate concentration (w/v)**	
	**0.09%**	**0.45%**
**Substrates**	**U/ml, (%)**	**U/ml, (%)**
Starch	1.2 (100)	1.6 (100)
Maltose	0.5 (42)	0.5 (31)
Maltotriose	2.0 (167)	0.9 (56)
Maltotetraose	1.4 (117)	0.6 (38)
Maltopentaose	1.5 (125)	0.4 (25)
Pullulan	ND*	< 0.01 (< 1)
Dextran	ND*	< 0.01 (< 1)
Isomaltotriose	ND*	< 0.01 (< 1)
Panose	ND*	< 0.01 (< 1)

The values represent the enzyme activity with starch and maltodextrins as substrates. The values in the parentheses represent the relative activity with its starch-hydrolyzing activity taken as 100%. Experiments were carried out in duplicate or triplicate, and the average values are indicated. *Not determined.

**Table 2:** Steady-state kinetic parameters of CauloGA at 30°C, pH 5.0

**Substrates**	***k***_**cat**_^**a **^**(s**^**-1**^**)**	***K***_**m**_^**a **^**(mM)**	***k***_**cat**_**/*K***_**m **_**(s**^**-1 **^**mM**^**-1**^**)**	***K***_**i**_^**b **^**(mM)**
Maltose	38.2 ± 0.94	0.87 ± 0.08	43.9	38.2 ± 1.11
Maltotriose	150 ± 4.37	0.16 ± 0.02	938	4.49 ± 0.31
Maltotetraose	155 ± 0.66	0.14 ± 0.004	1110	1.10 ± 0.06
Maltopentaose	122 ± 4.21	0.03 ± 0.008	4070	0.92 ± 0.04
Maltohexaose	125 ± 1.91	0.05 ± 0.004	2500	1.05 ± 0.08
Maltoheptaose	114 ± 4.57	0.04 ± 0.003	2850	1.25 ± 0.25

^a^*k*_cat_ and *K*_m_ values (means ± standard deviation) were obtained based on the results with maltose (0–4.5 mM), maltotriose (0–0.9 mM) and maltotetraose to maltoheptaose (0–0.36 mM) as the substrates. Experiments were performed in triplicate.

^b^*K*_i_ values (means ± standard deviation) were obtained based on the results with maltose to maltotetraose (0–18 mM) and with maltopentaose to maltoheptaose (0–9 mM) as the substrates. Experiments were carried out in triplicate.

Based on the kinetic data, the subsite affinities (*A*_i_) of CauloGA were evaluated according to the method of Hiromi et al. (1973), using Equation 1 and Equation 2.

(1) $$A_{n+1}=\mathrm{RT}\left\{\ln{\left(k_{\mathrm{cat}}/K_{m}\right)}_{n+1}-\ln{\left(k_{\mathrm{cat}}/K_{m}\right)}_{n}\right\}$$

(2) $${\left(k_{\mathrm{cat}}/K_{m}\right)}_{2}=0.018k_{\mathrm{int}}\exp\left\{{}^{\left(A_{1}+A_{2}\right)}{/}_{\mathrm{RT}}\right\}$$

where *A*_i_, R, T and *k*_int_ denote the subsite affinity (i: subsite number), gas constant, absolute temperature and intrinsic rate constant, respectively.

The subsite affinities, *A*_3_, *A*_4_, *A*_5_, *A*_6_ and *A*_7_ were estimated to be 7.7, 0.4, 3.2, -1.2 and -0.3 kJ/mol, respectively, from Eq. 1. The *A*_1_ + *A*_2_ value was estimated to be 27.8 kJ/mol from Eq. 2, by assuming *k*_int_ = (*k*_cat_)_2_.

## Discussion

In the Gram-negative bacterium *Caulobacter crescentus* genome, three putative maltodextrin-related hydrolase genes, *cc2282* (GA) (Nierman et al. 2001; Neugebauer et al. 2005; Lohmiller et al. 2008), *cc2285* and *cc2286* (α-amylases) are assigned in the *malA* gene cluster that encodes the maltodextrin transport system. However, the functional evidence for this assignment has been lacking. Therefore, the roles and functions of these putative glycoside hydrolase family proteins of *C. crescentus* still remain obscure. In the present work, we cloned the *cc2282* gene of *C. crescentus*, and identified it as the CauloGA gene based on the properties of the gene product that was expressed in *E. coli* as a protein fused with *Staphylococcus* Protein A at the N-terminus.

Based on the deduced amino acid sequence of the *cc2282* gene product, it is likely composed of an N-terminal domain and a catalytic domain. The putative catalytic domain of the *cc2282* gene product has five conserved regions, which is a common feature shared with other GAs in the literature. The structural characteristics described above are consistent with the notion that the *cc2282* gene represents the CauloGA gene. However, functional evidence to verify this notion has been lacking, since we were unable to detect the GA activity in either the culture medium or the cell homogenate of *C. crescentus* CB15. Moreover, it was reported that the *cc2282* gene product was not essential for the growth of *C. crescentus* on maltodextrins because inactivation of the *cc2282* gene by the Ω resistance cassette insertion did not reduce the growth rate of *C. crescentus* on maltose, maltotriose or maltotetraose (Lohmiller et al. 2008). The *cc2282* gene product may be dispensable in the presence of a sufficient concentration of maltodextrin in the medium. Expression of the gene product in functional form and biochemical characterization of the product is therefore necessary before the gene assignment is finally established.

Although it was difficult to express CauloGA in a soluble and active form in *E. coli,* we finally succeeded in this study in expressing CauloGA in the soluble and active form in *E. coli*. The pEZZ18 vector used in this study was originally designed to utilize the *Staphylococcus* Protein A leader sequence for the secretion of a fusion protein directly into the cell culture medium (Moks et al. 1987). However, in the present study, the fusion protein, ProteinA-CauloGA, was not secreted but was expressed intracellularly in the *E. coli* HB101 cells. The Protein A-CauloGA fusion protein was also expressed using the pET21 vector, although the expression was lower than that with the pEZZ18 system. This result may imply that having the Protein A moiety as a fusion partner aids in folding and solubilizing the CauloGA moiety. It may be argued that the fusion of the protein A moiety may somehow alter the catalytic properties of the native enzyme protein. However, we consider that this is unlikely, because the trace amount of CauloGA expressed without protein A fusion using the pCold I expression vector showed very similar characteristics with the Protein A-CauloGA fusion product with respect to the substrate specificity and temperature- and pH-dependence. The functional expressions as Protein A-fusion proteins without impairing original catalytic activities were also reported recently on keratinases, lipases and mammalian chitinase (Tiwary and Gupta 2010; Rajput et al. 2012; Kumari and Gupta 2012; Kashimura et al. 2013).

**Figure 7:**
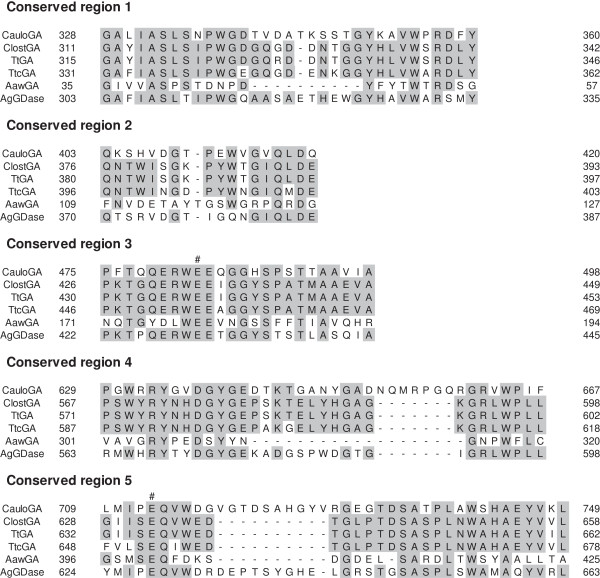
**Sequence alignments of the conserved regions (1–5) of CauloGA with other GAs and GDase.** The sequences aligned are: CauloGA, *C. crescentus* CB15 glucoamylase; ClostGA, *Clostridium* sp. G0005 glucoamylase; TtGA, *T. thermosaccharolyticum* glucoamylase; TtcGA, *T. tengcongensis* glucoamylase; AawGA, *A. awamori* glucoamylase; AgGDase, *A. globiformis* I42 glucodextranase. The symbol “#” denotes putative catalytic residues. Identical amino acid residues common to three or more of these enzymes are shaded.

We identified the *cc2282* gene product as GA (CauloGA) based on a number of characteristics that the gene product shares with other GAs; for instance, it produces glucose from maltotriose and starch, but not from α-(1 → 6) linkaged substrates such as dextran, indicating that *cc2282* gene product is not a GDase. Moreover, the released product was identified as the β-anomer of glucose. These results indicate that CauloGA is an exo-type hydrolytic enzyme with the inverting mechanism, that is, a GA. In addition, CauloGA is active in an acidic environment (pH 4.5-7.0). This is similar to other GAs that have conserved glutamic acid residues, that are involved in the hydrolysis as the general acid and base catalysts, in the conserved regions 3 and 5 of the catalytic domain (Sierks et al. 1990; Harris et al. 1993; Frandsen et al. 1994; Ohnishi et al. 1994). The two glutamic acid residues, Glu483 and Glu713, in the conserved regions 3 and 5 of the putative catalytic domain of CauloGA, located at positions corresponding to those conserved glutamic acid residues in other GAs (Figure 7), may be involved in the hydrolysis of the substrate similarly to those in other GAs. CauloGA has a strong preference for the substrates with α-(1 → 4)-glucosidic linkages, such as maltose, maltotriose and starch, to those with α-(1 → 6)-glucosidic linkages. It is noted that the optimal temperature of CauloGA was approximately 40°C, and irreversible heat-inactivation became noticeable above 40°C as shown in Figure 3. However, the enzyme seems to begin partially, though reversibly, inactivated at temperatures above 30°C. This is suggested by the observation that the Arrhenius plot was apparently linear below 30°C, but that deviation from linearity was noted above 30°C (Additional figure 4: Figure S4). For this reason, we carried out kinetic experiments at 30°C and pH 5.0.

When the kinetic constants of CauloGA, with maltotriose as a substrate, are compared with those of other GAs, *k*_cat_ was higher and *K*_m_ was lower, and consequently, the *k*_cat_/*K*_m_ value of CauloGA was the highest value among the GAs reported (Table 3). With maltose as a substrate, *k*_cat_ and *K*_m_ were slightly higher and lower, respectively, than other GAs. In addition, CauloGA had significantly higher affinities for maltodextrins composed of 5–7 glucose units than shorter maltodextrins (Table 2). The lower *K*_m_ values of CauloGA compared with other GAs may imply that the enzyme plays a role in the environmental conditions of *C. crescentus* inhabitation in which the availability of the substrates is usually limited. Subsite affinities *A*_1_ + *A*_2_, *A*_3_, *A*_4_ and *A*_5_ estimated from the kinetic parameters of CauloGA were positive, but *A*_6_ and *A*_7_ were negative. The values of *A*_1_ + *A*_2_, *A*_3_ and *A*_5_ imply a high affinity of the corresponding subsites to glucose residues of substrates. These subsite affinities may suggest that the CauloGA molecule provides five subsites for substrate binding, although we have to wait crystallographic elucidation of the enzyme structure for definite conclusion.

**Table 3:** Comparison of kinetic parameters of various GAs with maltose and maltotiose as the substrates

	**Substrates**						**Conditions**
	**Maltose**			**Maltotriose**			**Temp (°C), pH**
**Enzymes**	***k***_**cat **_**(s**^**-1**^**)**	***K***_**m (mM)**_	***k***_**cat**_**/*K***_**m **_**(s**^**-1**^ **mM**^**-1**^**)**	***k***_**cat **_**(s**^**-1**^**)**	***K***_**m **_**(mM)**	***k***_**cat**_**/*K***_**m **_**(s**^**-1**^ **mM**^**-1**^**)**	
CauloGA	38.2	0.87	43.9	150	0.16	938	30, 5.0
ClostGA	10.4	3.67	2.83	74.1	0.69	107	25, 4.5
TGA	16.9	0.10	167	76.4	0.38	201	40, 6.5
TtcGA	149	13.4	11.1	368	1.93	191	75, 5.0
AawGA	14.4	1.73	8.3	62	0.73	86	50, 4.4
AmyC	9.3	0.53	17.5	54.7	0.49	112	40, 5.5

The kinetic parameters of various GAs and the conditions for activity measurements are taken from the sources as follows: CauloGA (*C. crescentus* CB15 glucoamylase), this study; ClostGA (*Clostridium*; Ohnishi et al. 1992); TGA (*Thermoactinomyces vulgaris*; Ichikawa et al. 2004); TtcGA (*Thermoanaerobacterium tengcongensis*; Zheng et al. 2010); AawGA (*Aspergillus awamori*; Sierks et al. 1989); AmyC (*Rhizopus oryzae*; Mertens et al. 2010)

Another remarkable feature of CauloGA was the extensive apparent substrate inhibition by maltotriose at concentrations that were 10-fold higher than the *K*_m_ value. A similar but more pronounced inhibition was also observed with various maltodextrins as substrates, but the inhibition was only marginal with maltose as a substrate. In view of the report on fungal GA, AmyC, describing the inhibition resulting from the accumulation of enzyme-product intermediates (Mertens et al. 2010), the inhibition of CauloGA by substrates at high concentration ranges may actually be a result of the product inhibition rather than the substrate inhibition. With maltotriose and maltotetraose as substrates, we may suppose that the reaction products, maltose and maltotriose, respectively, were slowly released from CauloGA because of their high affinity, while glucose, the product of maltose hydrolysis, was released more rapidly. However, the precise mechanisms of this inhibition may be more complicated and remain to be elucidated in future studies.

Despite the similarity of the catalytic properties of CauloGA with other GAs, it is much less thermostable than other thermophilic GAs. The maltotriose-hydrolyzing activity of CauloGA was optimal at 40°C (Figure 3A), and the enzyme was labile at temperatures above 40°C (Figures 3B and S3). The thermolabile characteristics of CauloGA may be related to the insertions of approximately ten amino acid residues in both the putative N-terminal and the catalytic domains of CauloGA that make its molecular mass (approximately 82 kDa) slightly larger than other GAs. It is also interesting that amino acid composition was considerably different between CauloGA and other bacterial and archaeal thermophilic GAs. CauloGA contained more alanine (14.5%), glycine (10.2%) and threonine (7.8%) but less isoleucine (2.6%) asparagine (1.9%) and tyrosine (3.9%) than thermophilic GAs which are composed of less alanine (4.6-8.8%), glycine (5.3-8.7%) and threonine (3.9-4.9%), and more isoleucine (4.9-10.6%), asparagine (2.3-7.9%) and tyrosine (3.5-7.7%), respectively, although it is unclear at present how such differences are related to the thermolability of CauloGA. Such a trend was noted not only with the overall amino acid composition but also with the amino acid composition of the putative catalytic domain and of the conserved regions of GAs (Figure 7).

In conclusion, we were able to unambiguously demonstrate that the *cc2282* gene product represents CauloGA based on its catalytic properties. CauloGA is active at lower substrate concentrations and at lower temperatures compared with the thermophilic GAs reported. The mechanisms underlying the inhibition noted at elevated substrate concentration ranges and the structural basis of the thermolability should be investigated further. These studies would lead to a deeper insight into the relationships between the function and the structure of GA and GH15 family enzymes and also contribute toward the understanding of the glycoside hydrolase system of *C. crescentus*.

## Competing interests

The authors declare that the research was conducted in the absence of any commercial or financial relationships that could be construed as potential conflicts of interest.

## Supplementary Material

Additional file 1: Figure S1 The nucleotide sequence and the deduced amino acid sequence of CauloGA. The numbers in the right and left margins show the amino acid and base numbers, respectively, counted from the N-terminal position of the protein. Trm denotes a stop codon. The dashed underlined amino acid sequence is a putative signal peptide predicted by SignalP (http://www.cbs.dtu.dk/services/SignalP/). The underlined amino acid sequences with a number 1 to 5 at the left end of each line are sequences resembling the regions conserved among the GA family members. The two putative catalytic residues, Glu483 and Glu713, are shaded. Click here for file

Additional file 2: Figure S2 ^1^H NMR analysis of the products of maltotriose hydrolysis by CauloGA **(A)** and by α-glucosidase **(B)**. The times indicated (0, 30 and 60 min) represent the beginning of the spectral data acquisition, and peaks assigned to the α- (around 5.2 ppm) and β-anomers (around 4.6 ppm) are shown. Click here for file

Additional file 3: Figure S3 The fluorescence intensity versus temperature curve showing the temperature range in which the unfolding of CauloGA occurr. The experimental procedure was detailed in Materials and Methods. Experiments were carried out in duplicate and the average values are shown. Click here for file

Additional file 4: Figure S4 Arrhenius plot of the initial rate of CauloGA reaction toward maltotriose. The values were estimated from the results in Figure 3A. Click here for file
