# Supplementary figures and images for: Glucoamylase of Caulobacter crescentus CB15: cloning and expression in Escherichia coli and functional identification
Source: AMB Express. 2014 Jan 27;4:5. doi: 10.1186/2191-0855-4-5 (PMC3917699; doi:10.1186/2191-0855-4-5)

# Sakaguchi et al. Additional Figure S2

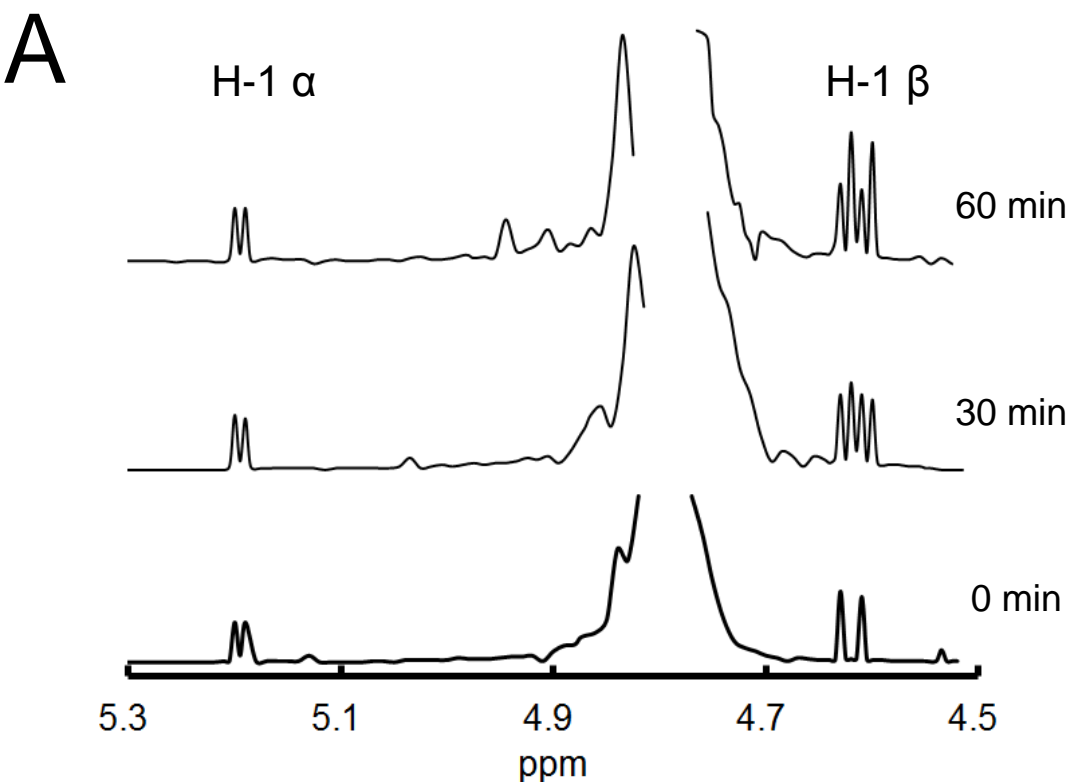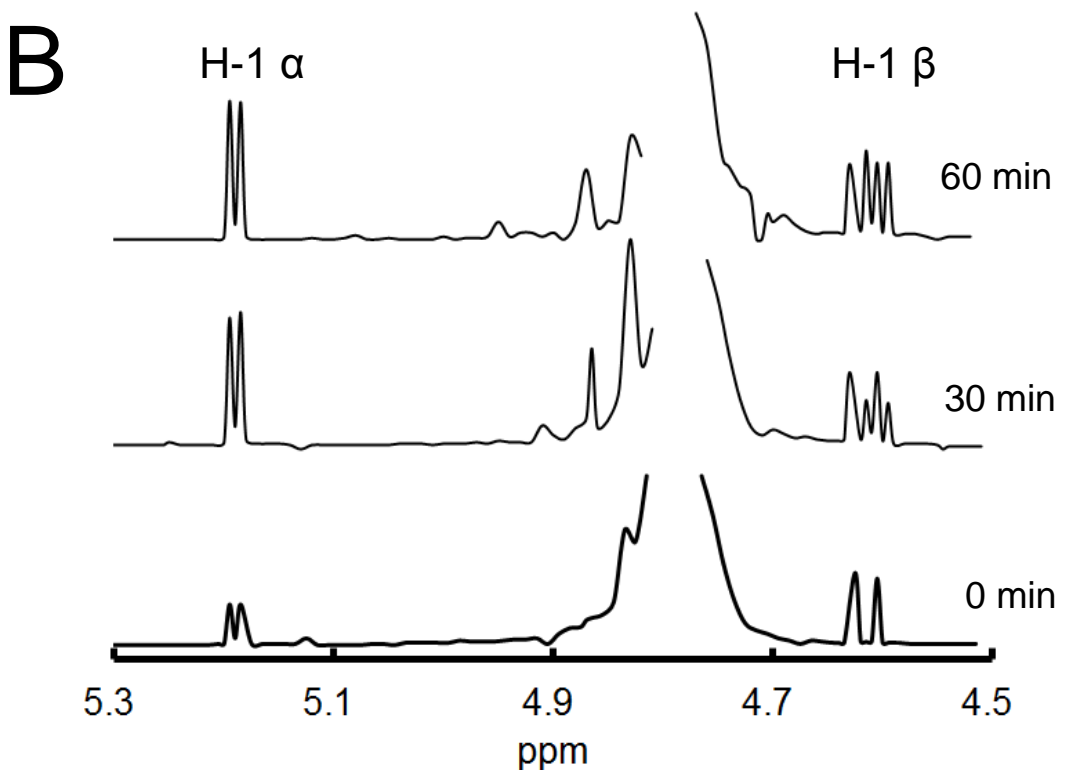

Supplement: Additional file 2: Figure S2 — 1H NMR analysis of the products of maltotriose hydrolysis by CauloGA (A) and by α-glucosidase (B). The times indicated (0, 30 and 60 min) represent the beginning of the spectral data acquisition, and peaks assigned to the α- (around 5.2 ppm) and β-anomers (around 4.6 ppm) are shown. [file 2191-0855-4-5-S2.pdf]

# Sakaguchi et al. Additional Figure S3

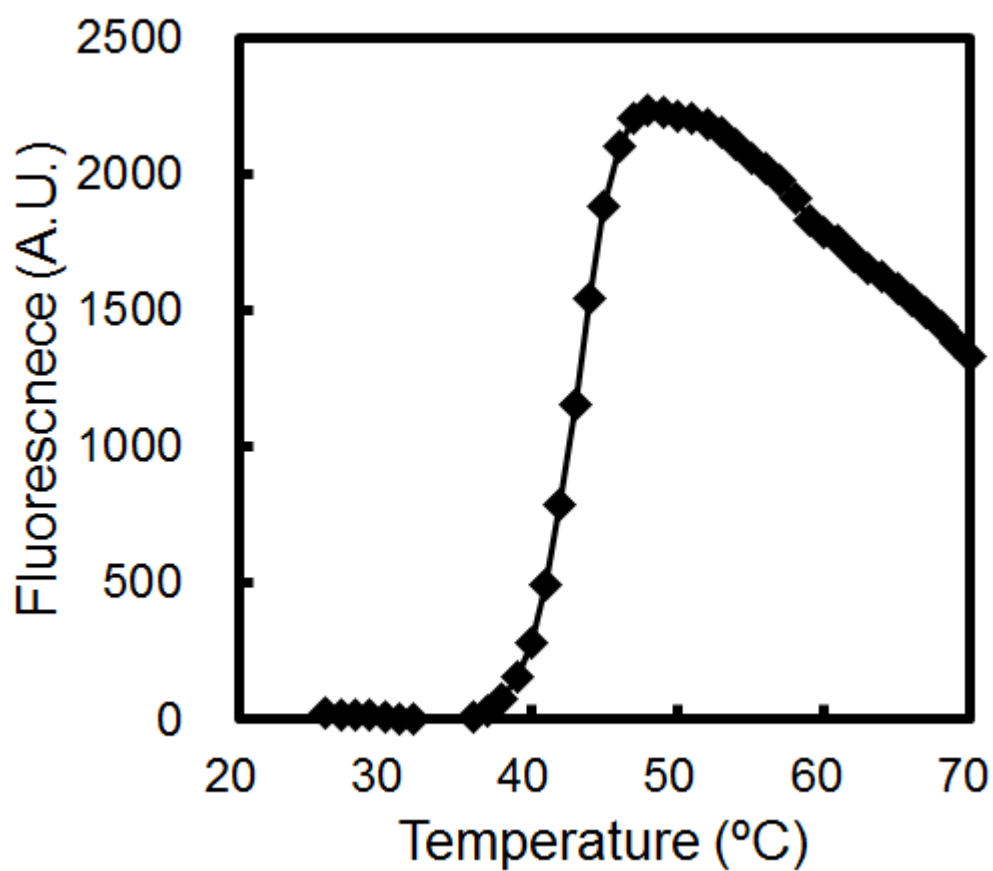

Supplement: Additional file 3: Figure S3 — The fluorescence intensity versus temperature curve showing the temperature range in which the unfolding of CauloGA occurr. The experimental procedure was detailed in Materials and Methods. Experiments were carried out in duplicate and the average values are shown. [file 2191-0855-4-5-S3.pdf]

# Sakaguchi et al. Additional Figure S4

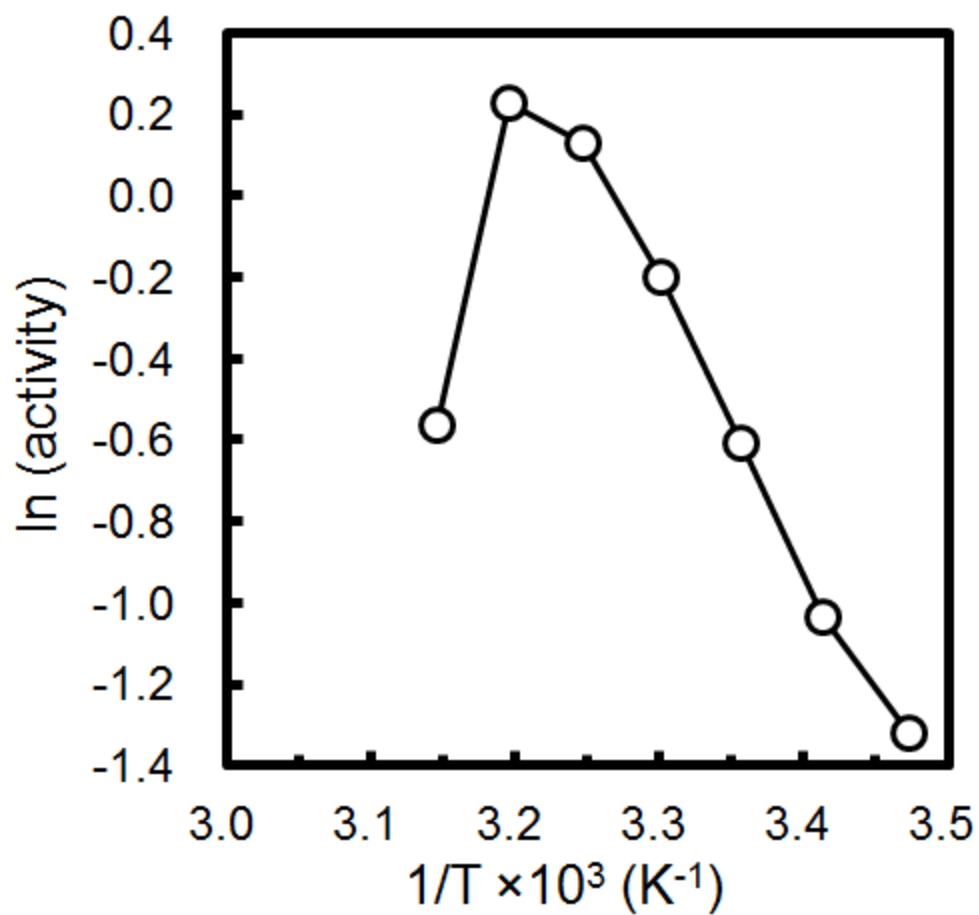

Supplement: Additional file 4: Figure S4 — Arrhenius plot of the initial rate of CauloGA reaction toward maltotriose. The values were estimated from the results in Figure 3A. [file 2191-0855-4-5-S4.pdf]
